# Quality-recommendation paradox in adult hypothyroidism guidelines: a systematic appraisal of methodological quality, recommendation consistency, and citation impact

**DOI:** 10.3389/fendo.2026.1998518

**Published:** 2026-10-30

**Authors:** Peng Zhi, Xiaohe Yan, Chaofan Zhang, Jing Zhao

**Affiliations:** 1 Department of General Practice, Henan Provincial Chest Hospital, Zhengzhou, China; 2 Department of Rehabilitation, Henan Provincial Chest Hospital, Zhengzhou, China

**Keywords:** AGREE II, citation impact, clinical practice guidelines, guideline appraisal, hypothyroidism, quality-recommendation paradox, recommendation consistency

## Abstract

**Background:**

Hypothyroidism is one of the most common endocrine disorders, yet its adult guidelines disagree on screening, on treatment thresholds for subclinical disease, and on combination therapy. Whether higher methodological quality yields more conservative, more actionable, or more influential guidance remains unclear.

**Methods:**

PubMed, Web of Science, Scopus, CNKI, and VIP were searched from January 1, 2010 to August 25, 2026. Eligible documents were clinical practice guidelines and standard documents for adult hypothyroidism, published in Chinese or English. Four raters assessed all included documents with the Appraisal of Guidelines for Research and Evaluation II (AGREE II) instrument. Domain scores, intraclass correlation coefficients (ICCs), recommendation consistency, version evolution, emerging-topic coverage, and citation impact were examined. The study was registered with the International Prospective Register of Systematic Reviews (PROSPERO; CRD420261487294).

**Results:**

Fifteen guidelines were included: 12 English and 3 Chinese documents (2012-2025). Eight of 15 reached an overall quality score of at least 5.5 and were recommended for use. BMJ 2019 (6.25), AACE/ATA 2012 (6.20), and ETA 2025 (6.10) scored highest, and the two Chinese primary care guidelines scored lowest (4.86 and 4.77). For the thyroid-stimulating hormone (TSH) 4–10 mIU/L gray zone, BMJ 2019 avoided routine treatment, whereas ETA 2013 (5.76) was more interventional. Version evolution was evidence-driven in the ETA series but simplification-driven in Chinese primary care documents, and coverage of artificial intelligence, laboratory standardization, and reference intervals was largely descriptive. Exploratory analysis showed only weak quality-citation correlation (Spearman rho = 0.368, n = 13). Average ICC(2,k) values ranged from 0.651 to 0.895 across domains.

**Conclusion:**

Mean overall quality ranged from 4.77 to 6.25, and only 8 of 15 (53%) earned an unqualified recommendation for use; the most rigorous documents were BMJ Rapid Recommendations, AACE/ATA 2012, and ETA 2025. Rigorous guidelines often acknowledge uncertainty, whereas less rigorous documents supply clearer directives. In gray-zone decisions such as TSH 4–10 mIU/L, clinicians should pair guideline appraisal with explicit evidence summaries and should not assume that the most decisive advice is the most reliable. Guideline developers should strengthen stakeholder involvement, applicability, and scheduled updating.

## 1 Introduction

Hypothyroidism is among the most common endocrine disorders encountered in clinical practice. Clinical practice guidelines are the mainstream mechanism for standardizing its management, and clinicians implicitly assume that guidelines developed through more rigorous methods provide more trustworthy recommendations. This assumption is under strain: adult hypothyroidism guidelines diverge on screening, on treatment thresholds for subclinical disease, and on levothyroxine plus liothyronine combination therapy. Although pregnancy-specific guidelines have been appraised with the Appraisal of Guidelines for Research and Evaluation II (AGREE II) instrument (1, 6), no previous study has applied AGREE II to adult guidelines outside pregnancy, including recent documents such as the 2025 European Thyroid Association guideline (2), and it remains unclear whether higher methodological quality yields more conservative, more actionable, or more influential guidance. This study therefore applied AGREE II to all eligible adult hypothyroidism guidelines, quantified inter-rater reliability, mapped recommendation consistency and version evolution, examined coverage of emerging topics, and tested whether methodological quality was associated with citation impact and with the conservativeness of recommendations in the thyroid-stimulating hormone (TSH) 4–10 mIU/L gray zone.

## 2 Methods

### 2.1 Design and registration

The present study constitutes a systematic guideline appraisal reported according to the Preferred Reporting Items for Systematic Reviews and Meta-Analyses (PRISMA) 2020 statement (3) and the PRISMA-S extension (4). The protocol was registered with the International Prospective Register of Systematic Reviews (PROSPERO) on August 24, 2026 (CRD420261487294) (5). Only published documents were used; because no patient-level data were involved, ethics approval was not required.

### 2.2 Search strategy

PubMed, Web of Science, Scopus, CNKI, and VIP were searched from January 1, 2010 to August 25, 2026. The start date was set at 2010 to capture contemporary adult guidance developed around current thyroid-function testing and treatment frameworks; the final set contained no guideline published before 2012. These databases returned 113, 72, 69, 21, and 20 records, respectively (295 in total). Search terms covered hypothyroidism, subclinical hypothyroidism, and myxedema, combined with guideline and clinical practice guideline terms. The complete search strategies are available in the **Supplementary Material**. No language restriction was applied during the search; only Chinese- and English-language documents proceeded to screening. Embase was excluded from the search because institutional access was unavailable.

### 2.3 Eligibility criteria

Eligible documents were clinical practice guidelines or systematically developed standard documents covering diagnosis, screening, or management of adult hypothyroidism. Documents were organized for synthesis according to guideline type (formal guideline or systematically developed standard document), evidence-grading system, issuing organization, language, and publication year.

The review question was framed in a PICO-like structure: population, adults with clinical or subclinical hypothyroidism; index documents, clinical practice guidelines and systematically developed standard documents; comparator, not applicable; and outcomes, AGREE II domain and overall quality scores, inter-rater reliability, recommendation consistency, version evolution, emerging-topic coverage, and citation impact. We included Chinese- or English-language full-text documents published between January 1, 2010 and August 25, 2026. Pregnancy-specific, pediatric, and congenital guidelines were excluded. Guidelines for other thyroid conditions, standalone position statements without formal recommendations, narrative reviews, primary studies, meeting abstracts, and documents without recommendations or full text were also excluded.

### 2.4 Screening

Screening was conducted in a structured electronic screening ledger. No dedicated systematic review platform (such as Covidence or Rayyan) or text-mining automation was used. Two reviewers (Peng Zhi and Jing Zhao) screened titles and abstracts independently. Potentially eligible documents were subsequently assessed in full text by both reviewers. Disagreements were resolved by a third reviewer (Xiaohe Yan) through discussion or adjudication. Reasons for exclusion were recorded using predefined categories in a screening ledger. Duplicate records were removed using DOI and PMID for English-language records and title, author, and year for Chinese-language records. Full-text exclusions were recorded in three stages: full-text review (27 records), candidate refinement (30 records), and final exclusion (8 records). **Figure 1** summarizes the screening flow, and **Supplementary Table S1** lists all 65 full-text exclusions.

**Figure 1:**
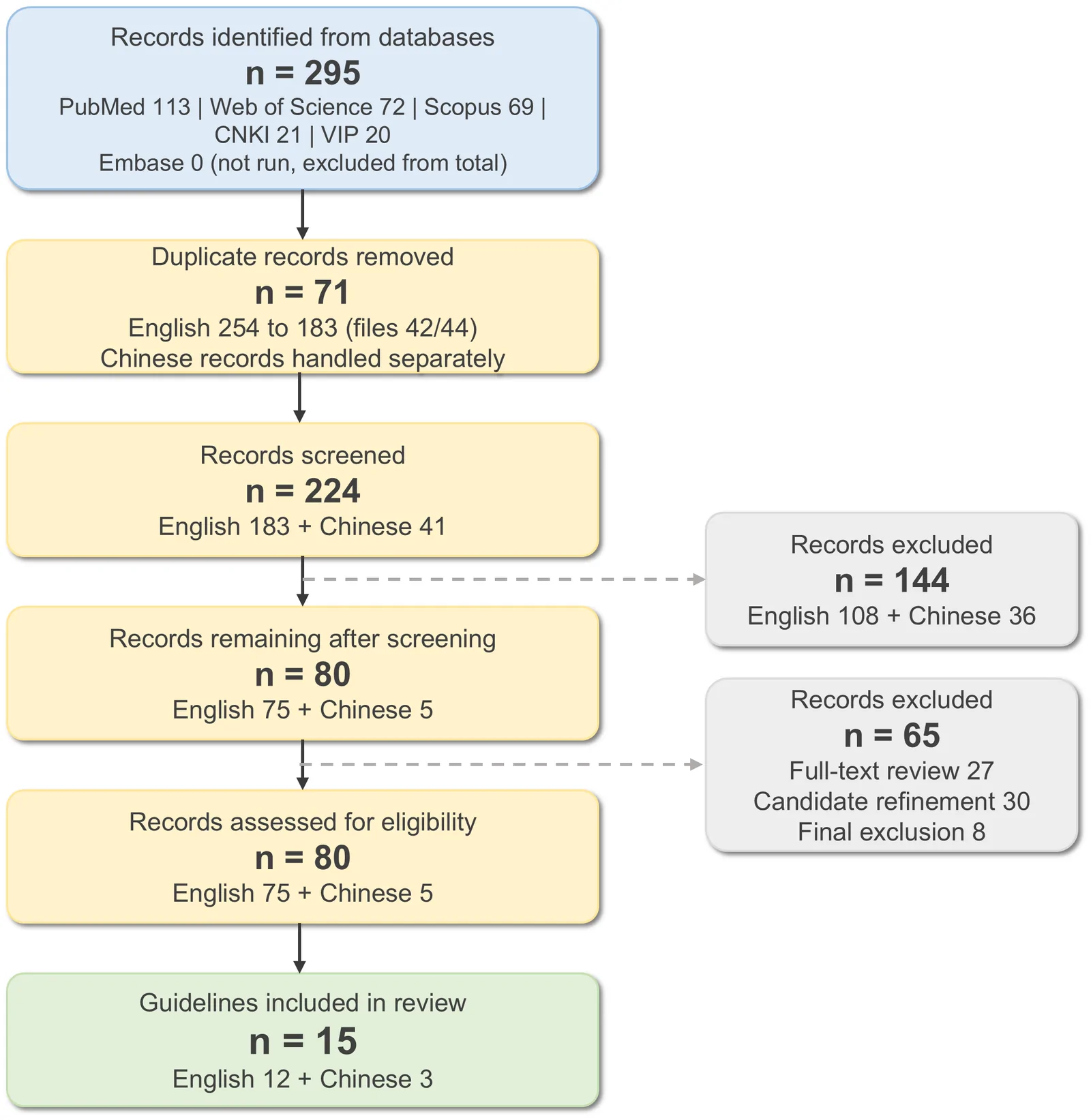
Preferred reporting items for systematic reviews and meta-analyses 2020 flow diagram of study identification, screening, eligibility assessment, and inclusion.

### 2.5 Data extraction

Data were extracted independently by two reviewers using a standardized form. For each guideline, the issuing organization, country, year, version, language, evidence system, key recommendations, recommendation strength, evidence levels, and coverage of emerging topics were recorded. Disagreements that arose during extraction were resolved with the third reviewer. Citation counts were retrieved on August 27, 2026 through DOI-based queries to OpenAlex, with Crossref as a fallback. Self-citations were retained because the analysis targets total scholarly impact as indexed by OpenAlex. Guidelines not indexed in these databases were recorded as not available.

Recommendation consistency was assessed across six predefined clinical topics selected *a priori* as the most contested issues in adult hypothyroidism management: screening strategy, treatment threshold for subclinical hypothyroidism, levothyroxine plus liothyronine combination therapy, thyroid-stimulating hormone (TSH) target in older adults, personalized TSH reference intervals, and management of subclinical hypothyroidism in infertile women. Two reviewers independently extracted each guideline’s position on each topic and classified it as recommended, conditional or individualized, not recommended, or not stated; disagreements were resolved by discussion with the third reviewer. Consistency was described narratively because the small number of guidelines precluded formal statistical comparison. Version evolution was assessed for guideline series with multiple versions (the European Thyroid Association series and the Chinese primary care series) by comparing recommendations on treatment thresholds and combination therapy across versions.

### 2.6 AGREE II appraisal

Four raters independently scored all 23 AGREE II items (6; **Supplementary File S3**) on a 1–7 scale after a training session and a calibration exercise on one guideline; discrepancies were discussed to align interpretation. The panel included one associate chief physician of general practice, two attending physicians of general practice, and one rehabilitation medicine supervisor nurse with methodological training. This composition was deliberate, because hypothyroidism is managed mainly in primary care. Judgments about applicability (D5) were therefore grounded in real-world use. The instrument comprises six domains: scope and purpose (D1, items 1-3), stakeholder involvement (D2, items 4-6), rigor of development (D3, items 7-14), and clarity of presentation (D4, items 15-17). Applicability (D5, items 18-21) and editorial independence (D6, items 22-23) complete the set. Standardized domain scores were computed as (obtained minus minimum possible) divided by (maximum minus minimum possible), multiplied by 100%. Minima and maxima were based on item counts and the four raters. Intraclass correlation coefficients (ICCs), reported as single-measure ICC(2,1) and average-measure ICC(2,k), were derived for each domain (7). Each rater also assigned an overall quality score (1-7), calculated as the mean of that rater’s 23 item scores, and the guideline-level overall quality score was the mean of the four raters’ scores. A guideline was classified as recommended for use when the mean overall score was at least 5.5, as uncertain when it was 4.0-5.49, and as not recommended when it was below 4.0.

### 2.7 Statistical analysis

Descriptive statistics and rankings were calculated for domain and overall scores. Inter-rater reliability used the two-way random-effects, absolute-agreement ICC, reported as single-measure ICC(2,1) and average-measure ICC(2,k) across four raters (8). ICC thresholds followed standard practice: below 0.50 poor, 0.50-0.75 moderate, 0.75-0.90 good, above 0.90 excellent. The association between overall quality and citation counts was examined using Spearman rank correlation. Subgroup comparisons by evidence system, organization type, language, and publication year were descriptive, and no causal inference was attempted. Analyses were performed using Python 3. A consolidated statistical verification report is provided in **Supplementary File S4**.

### 2.8 Emerging-topic analysis

An *ad hoc* framework, termed EndoCompass, was developed to classify coverage of emerging topics; it is not an established external instrument. An *a priori* keyword list covered artificial intelligence (machine learning, deep learning, algorithm, decision support) and laboratory standardization (standardization, harmonization, assay, calibration, quality control). Personalized reference intervals were covered by terms such as reference interval, age-specific, ethnicity, and personalized. Full texts were scanned electronically for the presence of these keywords. Each guideline was classified as having partial coverage or no coverage. Two reviewers independently verified the classifications against the full texts; disagreements were resolved by discussion.

## 3 Results

### 3.1 Included guidelines

After duplicate removal and screening, 15 of 295 identified records met the eligibility criteria (**Figure 1**). The final set included ETA 2025 (2), AACE/ATA 2012 (9), ATA 2014 (10), ETA 2013 (11), ETA 2018 (12), BMJ 2019 (13), Italian 2025 (14), and ASRM 2024 (15). BTA 2016 (16), Brazilian 2013 (17), ETA 2012 (L-T4+L-T3) (18), and the LT4/LT3 Consensus 2021 (19) were also included. CMA 2017 (20), Chinese Primary Care 2019 practice version (21), and Chinese Primary Care Medication 2021 (22) completed the list (**Table 1**).

**Table 1:** Standardized AGREE II domain scores and overall quality of the 15 included adult hypothyroidism guidelines.

Guideline	Year	D1%	D2%	D3%	D4%	D5%	D6%	Overall	Recommended
BMJ 2019 Rapid Recommendations	2019	87.5	84.7	90.6	86.1	88.5	79.2	6.25	Yes
AACE/ATA 2012	2012	86.1	79.2	90.6	86.1	89.6	77.1	6.20	Yes
ETA 2025 (levothyroxine monotherapy)	2025	90.3	76.4	86.5	86.1	86.5	79.2	6.10	Yes
ATA 2014	2014	87.5	73.6	83.3	83.3	82.3	77.1	5.92	Yes
ETA 2018 (central hypothyroidism)	2018	87.5	77.8	81.2	83.3	82.3	66.7	5.85	Yes
Italian 2025	2025	84.7	75.0	80.2	81.9	82.3	79.2	5.83	Yes
ETA 2013	2013	86.1	72.2	78.1	81.9	84.4	70.8	5.76	Yes
ASRM 2024	2024	79.2	79.2	75.0	77.8	79.2	66.7	5.58	Yes
Brazilian 2013	2013	77.8	66.7	73.4	76.4	70.8	70.8	5.37	Uncertain
CMA 2017	2017	87.5	65.3	69.8	76.4	66.7	66.7	5.29	Uncertain
LT4/LT3 Consensus 2021	2021	81.9	56.9	70.3	79.2	66.7	66.7	5.22	Uncertain
ETA 2012 (L-T4+L-T3)	2012	81.9	52.8	69.8	75.0	72.9	56.2	5.15	Uncertain
BTA 2016	2016	81.9	52.8	62.0	79.2	74.0	66.7	5.09	Uncertain
Chinese Primary Care 2019 (practice version)	2019	76.4	47.2	62.0	73.6	65.6	64.6	4.86	Uncertain
Chinese Primary Care Medication 2021	2021	68.1	56.9	61.5	65.3	63.5	64.6	4.77	Uncertain

Values are standardized domain scores (%). D1, scope and purpose; D2, stakeholder involvement; D3, rigor of development; D4, clarity of presentation; D5, applicability; D6, editorial independence. Overall, mean of the four raters’ overall quality scores (1-7). Recommended, mean overall quality score of at least 5.5; Uncertain, mean overall quality score 4.0-5.49; not recommended, mean overall quality score below 4.0.

### 3.2 Standardized domain scores and overall quality

Eight of 15 guidelines reached an overall quality score of at least 5.5 and were recommended for use. BMJ 2019 led at 6.25 (13), followed by AACE/ATA 2012 (6.20) (9) and ETA 2025 (6.10) (2). The two Chinese primary care guidelines scored lowest (4.86 and 4.77) (21, 22). **Table 1** and **Figure 2** present the full domain profile.

**Figure 2:**
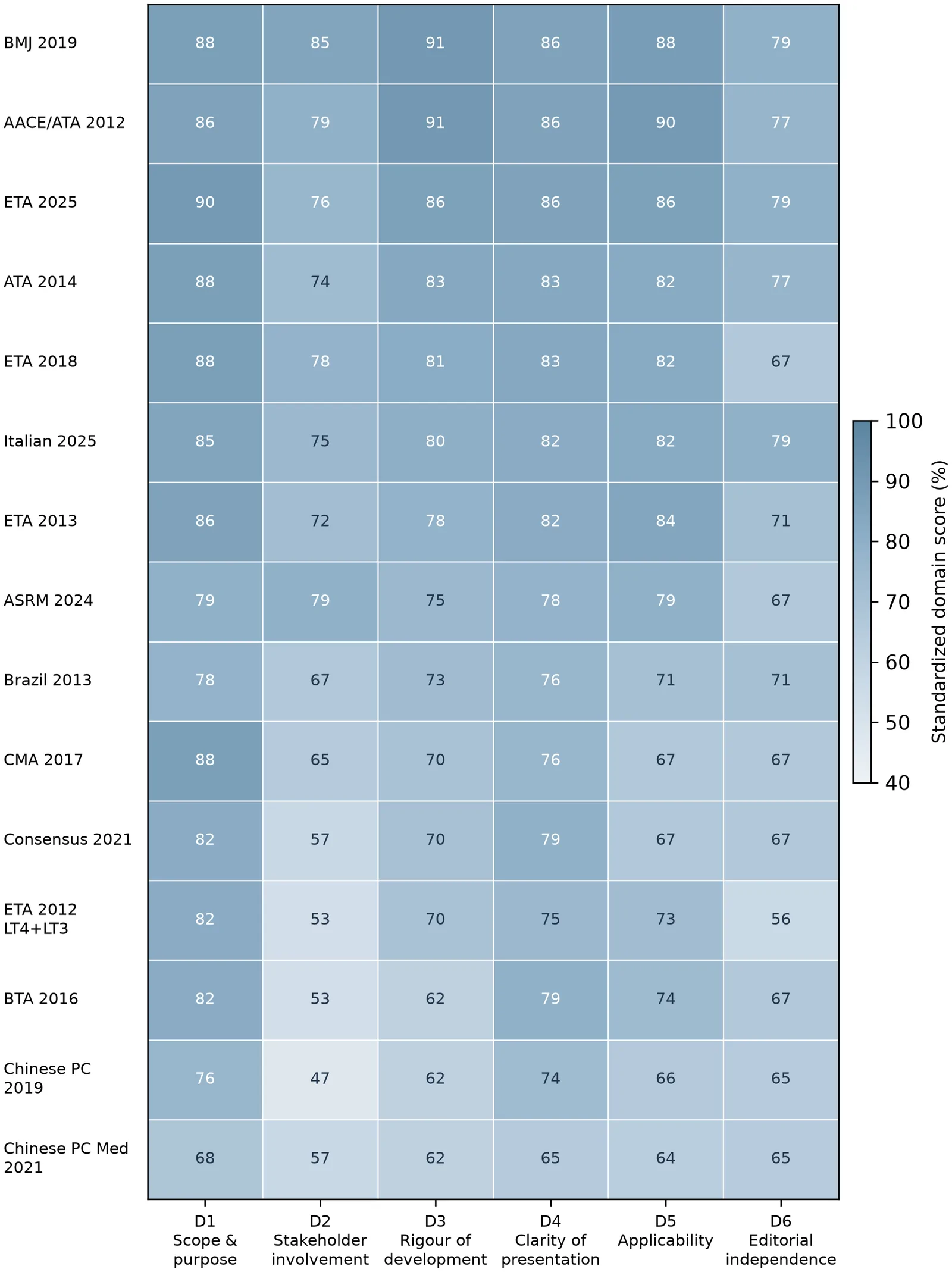
AGREE II standardized domain score heatmap of the 15 included adult hypothyroidism guidelines. Standardized scores range from 0% to 100%; darker colors indicate higher scores.

### 3.3 Inter-rater reliability

ICC(2,1) ranged from 0.318 (D1) to 0.680 (D2), and ICC(2,k) ranged from 0.651 to 0.895. Single-rater values for D1 (0.318) and D4 (0.348) fell below 0.50, the poor range. The four-rater averages were within the acceptable range (**Table 2**).

**Table 2:** Domain-specific intraclass correlation coefficients for inter-rater reliability across four raters.

Domain	ICC(2,1)	ICC(2,k)
D1 Scope and purpose	0.318	0.651
D2 Stakeholder involvement	0.680	0.895
D3 Rigor of development	0.567	0.840
D4 Clarity of presentation	0.348	0.681
D5 Applicability	0.547	0.828
D6 Editorial independence	0.417	0.741

ICC(2,1), single-measure intraclass correlation coefficient; ICC(2,k), average-measure intraclass correlation coefficient across four raters. ICCs were derived from a two-way random-effects, absolute-agreement model.

### 3.4 Recommendation consistency

Across the 15 guidelines, recommendations were more consistent for screening strategy and first-line treatment than for treatment thresholds. Most guidelines favored case finding over universal screening and identified levothyroxine monotherapy as standard replacement therapy. Diagnostic definitions were less heterogeneous than treatment decisions, although the evidence required to establish overt disease and the role of antibody testing were not reported uniformly.

Across the six predefined topics (**Figure 3**), the main disagreements concerned subclinical hypothyroidism. Most guidelines reserved treatment for thyroid-stimulating hormone (TSH) ≥10 mIU/L or individualized the 4–10 mIU/L range; ETA 2013 recommended a more active approach in younger or TPO-antibody-positive patients, whereas BMJ 2019 was explicitly conservative. Policies on levothyroxine plus liothyronine (LT4+LT3) also differed: ATA 2014, ETA 2025, and the Chinese primary care guidelines advised against routine use, whereas ETA 2012 and the LT4/LT3 consensus permitted conditional use in selected patients. ASRM 2024 set a lower treatment threshold for infertile women (TSH 2.5-4.0 mIU/L) than the threshold used for the general population. Treatment targets for older adults and personalized reference intervals were mostly individualized but varied in operational detail. These contrasts rest on a small set of guidelines and require confirmation in larger samples.

**Figure 3:**
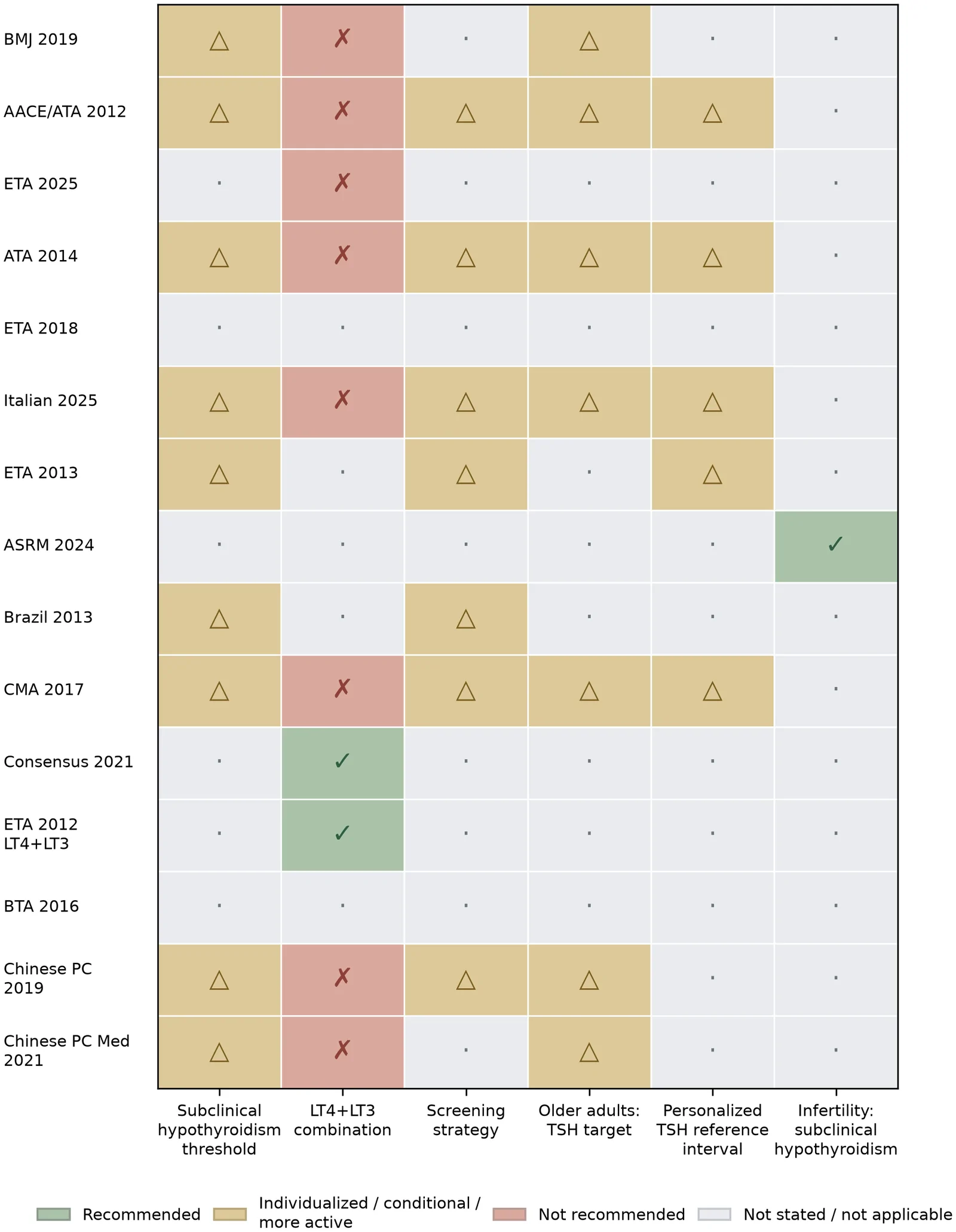
Recommendation consistency matrix across six predefined clinical topics (subclinical hypothyroidism treatment threshold, LT4 plus LT3 combination therapy, screening strategy, TSH target in older adults, personalized TSH reference interval, and subclinical hypothyroidism in infertile women) for the 15 included guidelines. Symbols: ✓, recommended; △, individualized, conditional, or more active; ✗, not recommended; ·, not stated or not applicable.

### 3.5 Version evolution

Version changes within the ETA and Chinese series were modest but clinically meaningful (**Figure 4**). In the ETA series, the 2025 guideline replaced conditional acceptance of LT4+LT3 with explicit monotherapy, a shift driven by new evidence. In the Chinese series, the 2019 and 2021 primary care versions simplified the threshold to TSH ≥10 mIU/L. Medication management and referral pathways were also refined in these versions.

**Figure 4:**
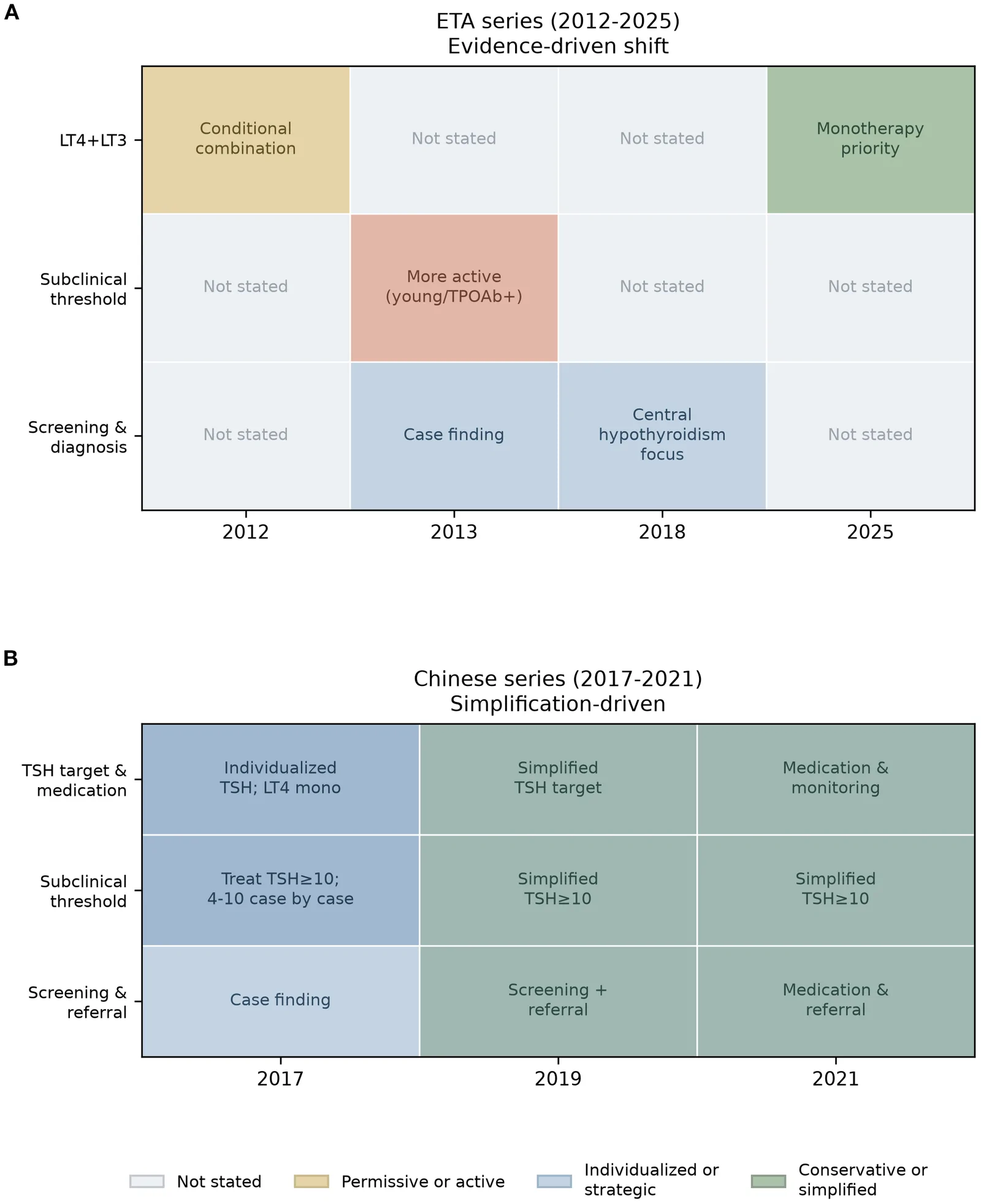
Version evolution matrix of **(A)** the European Thyroid Association and **(B)** the Chinese guideline series, showing changes in combination therapy, treatment thresholds, and screening or referral strategies over time. Cell colors indicate the nature of each version recommendation: gray, not stated; gold or clay, permissive or active; blue, individualized or strategy-oriented; green, conservative, simplified, or operational.

### 3.6 Descriptive observations

Guidelines built on the Grading of Recommendations, Assessment, Development and Evaluations (GRADE) framework or other systematic processes achieved higher rigor scores. In this sample, mean scores were higher for English-language guidelines than for Chinese-language guidelines, but this comparison was descriptive and was not designed to estimate a language effect. The largest gaps were observed in stakeholder involvement and applicability. Formal guidelines also outperformed consensus and recommendation documents, mainly on rigor.

### 3.7 Language-stratified comparison

In this descriptive comparison, the 12 English-language guidelines outscored the 3 Chinese guidelines on every domain. Domain means were 84.4% vs 77.3% (D1), 70.6% vs 56.5% (D2), 78.4% vs 64.4% (D3), 81.4% vs 71.8% (D4), 80.0% vs 65.3% (D5), and 71.4% vs 65.3% (D6). The largest absolute gaps appeared in applicability (14.7 points), stakeholder involvement (14.1), and rigor of development (14.0). Mean overall quality was 5.69 for English and 4.97 for Chinese guidelines. With only three Chinese guidelines, these differences are descriptive and may reflect sampling imbalance more than language itself.

### 3.8 EndoCompass coverage

Using the EndoCompass framework (an *ad hoc* emerging-topic coverage framework; Section 2.8), full-text scanning found AI or machine learning mentions in 10 of 15 guidelines. Laboratory standardization terms appeared in 10 of 15, and personalized reference interval terms in 14 of 15 (**Figure 5**). Most mentions were passing references rather than implementation-oriented guidance for practice.

**Figure 5:**
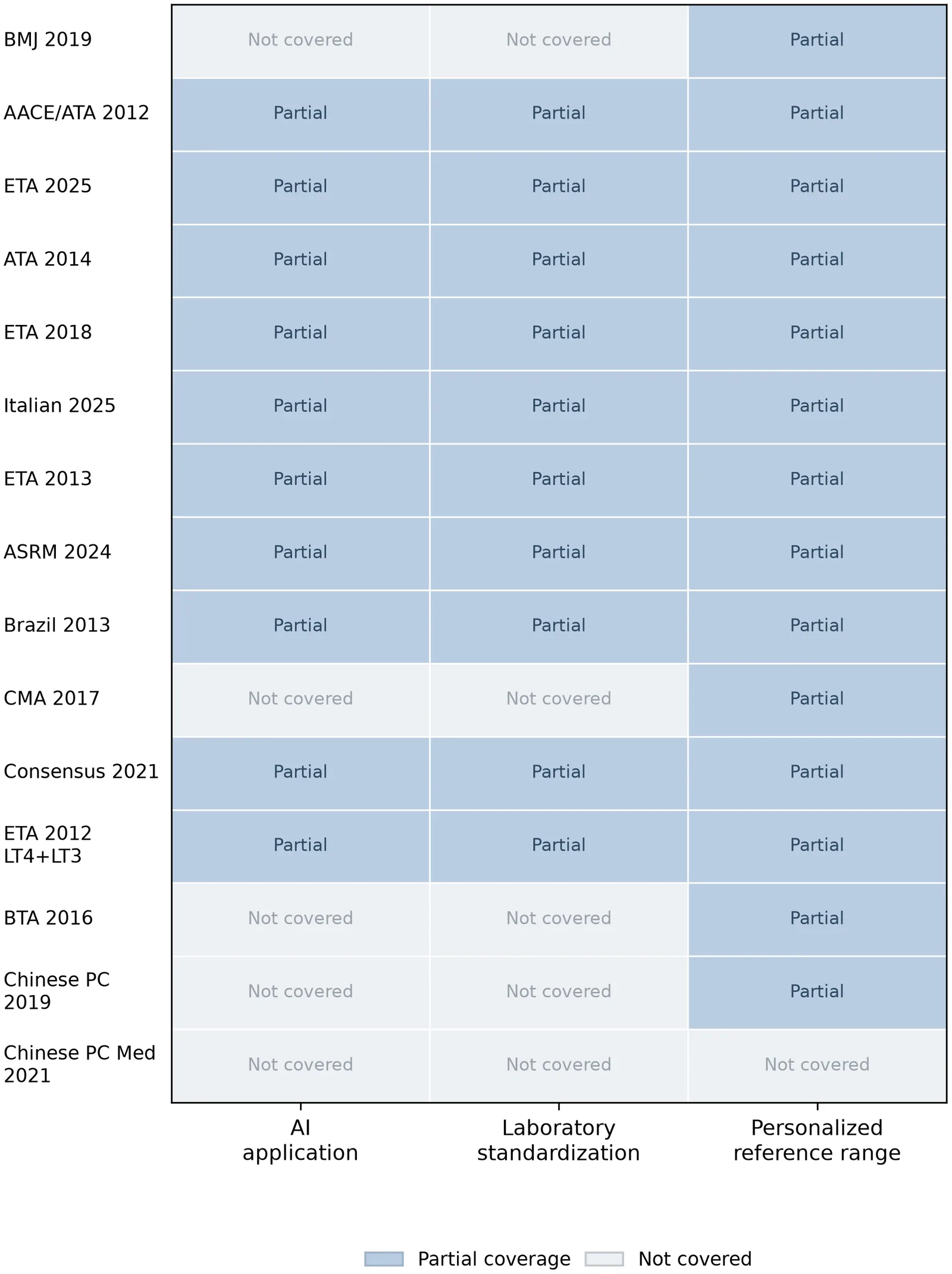
EndoCompass (emerging-topic coverage) heatmap of artificial intelligence, laboratory test standardization, and personalized reference intervals across the 15 included guidelines. The EndoCompass framework is defined in Section 2.8. Blue indicates partial coverage; gray indicates no coverage.

### 3.9 External anchoring: methodological quality and citation counts

Citation data were available for 13 of the 15 guidelines. OpenAlex supplied counts on August 27, 2026, with Crossref as a fallback. The two Chinese primary care guidelines were not indexed in international databases. Citations ranged from 3 (Italian 2025) to 1,896 (ATA 2014), with a median of 171. BMJ 2019, the highest-scoring guideline at 6.25, had 234 citations. The Spearman correlation between overall quality and citation count was weak to moderate (rho = 0.368; n = 13; **Figure 6**). Because citation counts are strongly influenced by publication age, journal visibility, and clinical controversy, this analysis was exploratory and is reported only to caution against using citation counts as a proxy for guideline quality.

**Figure 6:**
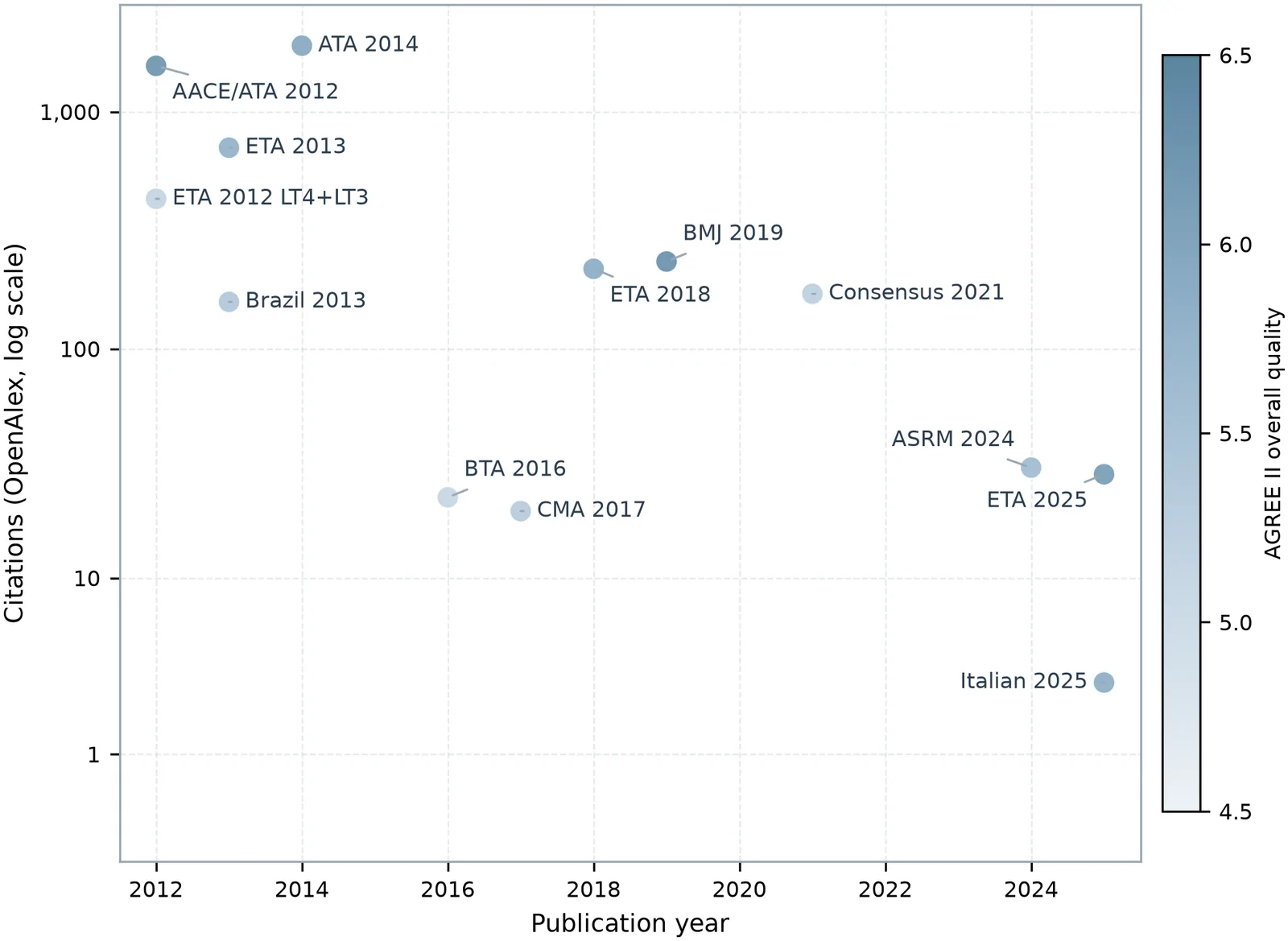
Citation counts (OpenAlex, retrieved August 27, 2026; log scale) by publication year; point color indicates the AGREE II mean overall quality score, and darker colors indicate higher scores (exploratory Spearman rho = 0.368, n = 13). Key points are labeled with their guideline abbreviations. Two Chinese primary care guidelines were not indexed in international citation databases and are excluded.

## 4 Discussion

To the best of the authors’ knowledge, this is the first systematic AGREE II appraisal of adult hypothyroidism guidelines outside pregnancy. Mean domain scores ranged from 67.8% (stakeholder involvement) to 83.0% (scope and purpose), and only scope and purpose exceeded 80%. The most consequential pattern was a quality-recommendation paradox, in which the most rigorous guidelines gave conservative, conditional advice and lower-quality documents gave more decisive directives. GRADE-based guidelines achieved higher rigor scores (23), consistent with earlier AGREE II appraisals (1). These findings imply that guideline users should evaluate process quality alongside decisiveness and that developers should prioritize stakeholder engagement, applicability, and scheduled updating.

These results extend earlier AGREE II work in two ways. Fang et al. (1) appraised pregnancy-specific hypothyroidism guidelines and identified stakeholder involvement and applicability as the weakest domains; the same profile emerged here among adult guidelines outside pregnancy. The present study also mapped recommendations to six contested clinical questions, traced version evolution, and examined citation impact. Unlike earlier appraisals, which focused on domain scores, this analysis identified an inverse pattern between methodological rigor and recommendation decisiveness. That pattern requires confirmation and should not be interpreted causally.

The AACE/ATA 2012 D3 score of 90.6% warrants comment because it is unusually high for a guideline from that era. D3 covers eight items, from evidence search to updating, and four raters scored them on a 1–7 scale. 90.6% corresponds to roughly 206 of 224 points, an average near 6.4 per item. The document followed an explicit evidence-based process, which explains the score. Its durability among the top-ranked guidelines suggests that process matters more than recency.

BMJ 2019 also reached 90.6% on D3, slightly above ATA 2014 (83.3%). The difference can be attributed to the Rapid Recommendations process. Guidelines are linked to updated systematic reviews, use a transparent GRADE evidence-to-decision framework, and commit to updating. ATA 2014 was rigorous in a more conventional, society-based way. The seven-point difference reflects how fully the evidence pathway was documented, not a judgment that one guideline is clinically better. This distinction matters for users: a higher D3 score does not necessarily mean better clinical recommendations.

Chinese primary care guidelines need more than a ranking to be understood. They function as simplified operational manuals for community physicians, not comprehensive evidence textbooks. Their lower scores on rigor, stakeholder involvement, and applicability may reflect that trade-off. Adding implementation tools, stakeholder engagement, and explicit update procedures would raise their AGREE II scores without changing their primary care role. Future updates could retain simplicity while adding an evidence appendix. This positioning does not exempt the documents from AGREE II standards. The instrument appraises development and reporting quality regardless of intended audience. Even a deliberately simplified manual should document stakeholder engagement and applicability.

Because only three Chinese guidelines were included, the language-stratified results should be interpreted as descriptive. Differences may be confounded by guideline type, care setting, and sample size, and they do not establish a language effect.

Averaged across four raters, reliability was moderate to good (ICC(2,k) 0.651-0.895). Single-rater ICCs for D1 (0.318) and D4 (0.348) were poor, a reminder that scope and clarity judgments are subjective. Raters must judge how explicitly objectives, health questions, and target populations are stated. They must also judge whether key recommendations are easy to identify. Long guidelines that present recommendations in multiple formats may invite disagreement. Multiple raters, standardized training, and calibration remain the sensible remedy. Earlier AGREE II work reached a similar conclusion about rater training (1).

The multidisciplinary composition of the rating panel was a deliberate strength. Because hypothyroidism is mostly managed in primary care, general practice clinicians and a methodologically trained rehabilitation nurse were included. The panel composition kept applicability judgments grounded in real-world practice. It may also explain part of the inter-rater variation in interpretive domains.

The recommendation data suggest a quality-recommendation paradox worth testing formally. The term describes an observation: higher methodological rigor accompanied more conservative recommendations, whereas lower-quality documents supplied more decisive directives. It does not imply a logical contradiction or a causal relationship. GRADE-based guidelines such as BMJ 2019 make uncertainty explicit, whereas consensus documents often resolve uncertainty through expert opinion. Clinicians should therefore distinguish decisiveness from trustworthiness: conditional recommendations can reflect a more transparent appraisal of evidence, whereas firm recommendations may rest on weaker synthesis. Critical appraisal of both methodological quality and the underlying evidence remains essential.

Two findings did not fit a simple quality-begets-impact model. ATA 2014 was the most cited guideline but not the highest scoring, and several high-quality guidelines left the TSH 4–10 mIU/L range unspecified while lower-quality primary care documents provided operational thresholds. These observations suggest that citation and clinical uptake may be shaped by controversy, accessibility, and decisiveness as well as methodological quality. They deserve direct investigation in larger samples.

The observed clinical heterogeneity carries practical consequences for everyday practice. Case finding dominated screening recommendations, but treatment thresholds for the TSH 4–10 mIU/L range split the field. Policies on LT4+LT3 ranged from routine avoidance to conditional use. ASRM 2024 set a lower threshold for infertile women, appropriate for that population. This choice illustrates how recommendations can diverge across different populations. Clinicians need clearer reporting of the evidence behind each recommendation, and international harmonization would help.

The ETA and Chinese guideline series appear to follow different updating models. The ETA series updated in response to new evidence, with the 2025 guideline shifting from conditional combination therapy to explicit monotherapy. The Chinese series updated primarily for usability, consolidating treatment thresholds across the 2019 and 2021 primary care versions and refining medication and referral pathways. This contrast supports a stratified guideline-development model: international societies may prioritize evidence-driven cycles that expose uncertainty, whereas primary care documents may prioritize simplification and operational clarity. The model yields a testable prediction: conditional recommendations from rigorous guidelines should remain more stable than decisive directives from consensus documents when new evidence emerges.

Coverage of emerging topics across the included guidelines was mostly descriptive. Ten of 15 guidelines mentioned AI or machine learning, 10 mentioned laboratory standardization, and 14 mentioned personalized reference intervals. Few of these documents translated the topics into implementation guidance. Guideline developers should move from topic mentions to concrete, actionable content. Fixed review cycles, for example every three to five years, would allow new evidence to enter systematically.

### Strengths and limitations

Strengths. This study used a systematic search across international and Chinese databases, with transparent screening and a complete exclusion ledger. Four trained raters with documented backgrounds completed a calibration exercise before formal scoring. The analysis linked methodological quality to recommendation consistency, version evolution, and emerging-topic coverage, providing a multidimensional view of guideline quality rather than a single score.

Limitations. Several limitations should be acknowledged. Eligibility was restricted to Chinese- and English-language documents, which may introduce language bias. Embase was not searched because institutional access was unavailable; PubMed, Web of Science, and Scopus overlap for English-language guidelines. Pregnancy-specific guidelines were excluded, and the boundary between guidelines and consensus documents is partly subjective. AGREE II assesses process and reporting quality rather than the clinical validity of recommendations, and no patient-outcome or adherence data were available to link higher quality to better outcomes. Subgroup comparisons remained descriptive because of small sample sizes. ICC values, particularly single-rater estimates for D1 and D4, warrant caution. Finally, emerging-topic coverage was assessed by keyword scanning rather than full qualitative synthesis, and the findings should be interpreted as an initial mapping. Patient and public representatives were not included in the appraisal panel. Although AGREE II does not require this for appraisal, patient perspectives would add value for preference-sensitive decisions, and future guideline appraisals should incorporate patient partners.

### Implications for guideline development and implementation

AGREE II does not measure whether recommendations respond to contested clinical questions. A content-methodology disconnect matrix (**Supplementary Table S2**) was constructed to examine this gap. High-scoring guidelines sometimes left the TSH 4–10 mIU/L gray zone as “individualized” without operational criteria, whereas lower-scoring primary care guidelines gave clear thresholds. If replicated, this disconnect argues for requiring updates to address the most contested questions directly, not merely to improve process. Low D5 scores point to a second gap: follow-up frequency and referral pathways are often missing. Beyond content, the format of guidelines also affects implementation. Future guidelines could be built for digital execution, following FAIR principles (findable, accessible, interoperable, reusable). Embedding guidelines in electronic health record decision support would extend their reach beyond PDF distribution. These implications operate at three levels. Clinicians should treat AGREE II scores as an indication of process quality, not as proof that a recommendation is clinically correct. Guideline developers should pre-specify how contested questions will be addressed, document stakeholder input, and include implementation, monitoring, and referral tools. Policymakers and digital health systems could require machine-readable recommendations and explicit update triggers to make guideline quality visible at the point of care.

### Future research

Future research should proceed in four directions. First, larger multilingual appraisals should test whether the inverse association between methodological quality and recommendation decisiveness persists. Second, longitudinal studies should examine whether conditional recommendations from high-quality guidelines remain stable when evidence updates. Third, implementation studies should compare clinician adherence to conditional versus decisive recommendations and link adherence to patient outcomes. Fourth, guideline-development research should evaluate whether patient-partner involvement, plain-language summaries, and digital decision-support tools improve uptake and reduce unwarranted variation.

## 5 Conclusion

Mean overall quality ranged from 4.77 to 6.25 across the 15 adult hypothyroidism guidelines, and only 8 of 15 (53%) earned an unqualified recommendation for use. The most rigorous documents (BMJ Rapid Recommendations, AACE/ATA 2012, and ETA 2025) often gave conservative, conditional advice, whereas less rigorous documents supplied more decisive directives, a pattern we term the quality-recommendation paradox. Clinicians should not rely on publication date, issuing society, or citation counts as proxies for quality, and should interpret gray-zone recommendations (TSH 4–10 mIU/L) with caution. Developers should strengthen stakeholder engagement, implementation tools, follow-up and referral pathways, and scheduled updating. Future research should test whether methodological quality predicts recommendation stability and whether clinicians adhere to conditional recommendations as readily as to decisive directives.

## Data Availability

The original contributions presented in the study are included in the article/**Supplementary Material**. Further inquiries can be directed to the corresponding author. The raw AGREE II rating sheets are available from the corresponding author upon reasonable request.

## Supplementary material

Supplementary Table 1 Full-text exclusion ledger of 65 records, with stage, reason, and source file for each excluded record. The ledger comprises 27 full-text review exclusions, 30 candidate refinement exclusions, and 8 final exclusions. Stage and exclusion codes follow the screening methods in Section 2.4; the English version is provided as a separate CSV file.

Supplementary Table 2 Content-methodology disconnect matrix of overall methodological quality and responsiveness to the thyroid-stimulating hormone (TSH) 4–10 mIU/L gray zone across the 15 included guidelines. Overall quality scores are the four-rater means presented in **Table 1**. Responsiveness to the TSH 4–10 mIU/L gray zone was classified as explicit, partially explicit, not stated, or not applicable on the basis of the finalized recommendation matrix (Section 3.4); classifications are exploratory.
