# Supplementary material for: Quality-recommendation paradox in adult hypothyroidism guidelines: a systematic appraisal of methodological quality, recommendation consistency, and citation impact
Source: Front Endocrinol (Lausanne). 2026 Oct 30;17:1998518. doi: 10.3389/fendo.2026.1998518 (PMC13759808; doi:10.3389/fendo.2026.1998518)
Supplement: Supplementary file 1 [file DataSheet1.zip › Supplementary_Reporting_S6_References.docx]

# References (Vancouver; final numbering 2026-08-28)

Numbering follows the order of first appearance in the manuscript (file 81). The guideline and prior-study references were verified against PubMed on 2026-08-26; methodology references are standard citations; three Chinese guidelines are cited with English translated titles and the [in Chinese] marker, and all three have DOIs.

Format notes: BMJ 2021 uses the article number n71 (PubMed: 2021;372:n71); BMJ 2019 uses l2006. Reference 5 is the PROSPERO registration CRD420261487294.

## References

1. Fang Y, Yao L, Sun J, Zhang J, Li Y, Yang R, et al. Appraisal of clinical practice guidelines on the management of hypothyroidism in pregnancy using the Appraisal of Guidelines for Research and Evaluation II instrument. Endocrine. 2018;60(1):4-14. doi:10.1007/s12020-018-1535-2

2. Centanni M, Duntas L, Feldt-Rasmussen U, Koehrle J, Peeters RP, Razvi S, et al. ETA guidelines for the use of levothyroxine sodium preparations in monotherapy to optimize the treatment of hypothyroidism. Eur Thyroid J. 2025;14(4):e250123. doi:10.1530/ETJ-25-0123

3. Page MJ, McKenzie JE, Bossuyt PM, Boutron I, Hoffmann TC, Mulrow CD, et al. The PRISMA 2020 statement: an updated guideline for reporting systematic reviews. BMJ. 2021;372:n71. doi:10.1136/bmj.n71

4. Rethlefsen ML, Kirtley S, Waffenschmidt S, Ayala AP, Moher D, Page MJ, et al. PRISMA-S: an extension to the PRISMA statement for reporting literature searches in systematic reviews. Syst Rev. 2021;10(1):39. doi:10.1186/s13643-020-01542-z

5. Centre for Reviews and Dissemination. PROSPERO: International prospective register of systematic reviews [Internet]. York: University of York; 2026 [cited 2026 Aug 27]. Available from: https://www.crd.york.ac.uk/PROSPERO/view/CRD420261487294

6. Brouwers MC, Kho ME, Browman GP, Burgers JS, Cluzeau F, Feder G, et al. AGREE II: advancing guideline development, reporting and evaluation in health care. CMAJ. 2010;182(18):E839-E842. doi:10.1503/cmaj.090449

7. Shrout PE, Fleiss JL. Intraclass correlations: uses in assessing rater reliability. Psychol Bull. 1979;86(2):420-428. doi:10.1037/0033-2909.86.2.420

8. Koo TK, Li MY. A guideline of selecting and reporting intraclass correlation coefficients for reliability research. J Chiropr Med. 2016;15(2):155-163. doi:10.1016/j.jcm.2016.02.012

9. Garber JR, Cobin RH, Gharib H, Hennessey JV, Klein I, Mechanick JI, et al. Clinical practice guidelines for hypothyroidism in adults: cosponsored by the American Association of Clinical Endocrinologists and the American Thyroid Association. Endocr Pract. 2012;18(6):988-1028. doi:10.4158/EP12280.GL

10. Jonklaas J, Bianco AC, Bauer AJ, Burman KD, Cappola AR, Celi FS, et al. Guidelines for the treatment of hypothyroidism: prepared by the American Thyroid Association task force on thyroid hormone replacement. Thyroid. 2014;24(12):1670-1751. doi:10.1089/thy.2014.0028

11. Pearce SHS, Brabant G, Duntas LH, Monzani F, Peeters RP, Razvi S, et al. 2013 ETA guideline: management of subclinical hypothyroidism. Eur Thyroid J. 2013;2(4):215-228. doi:10.1159/000356507

12. Persani L, Brabant G, Dattani M, Bonomi M, Feldt-Rasmussen U, Fliers E, et al. 2018 European Thyroid Association (ETA) guidelines on the diagnosis and management of central hypothyroidism. Eur Thyroid J. 2018;7(5):225-237. doi:10.1159/000491388

13. Bekkering GE, Agoritsas T, Lytvyn L, Heen AF, Feller M, Moutzouri E, et al. Thyroid hormones treatment for subclinical hypothyroidism: a clinical practice guideline. BMJ. 2019;365:l2006. doi:10.1136/bmj.l2006

14. Guglielmi R, Mian C, Novizio R, Paoletta A, Persichetti A, Samperi I, et al. Italian guidelines for the management of adult individuals with primary hypothyroidism outside pregnancy. J Endocrinol Invest. 2025;48(10):2295-2313. doi:10.1007/s40618-025-02652-y

15. Practice Committee of the American Society for Reproductive Medicine. Subclinical hypothyroidism in the infertile female population: a guideline. Fertil Steril. 2024;121(5):765-782. doi:10.1016/j.fertnstert.2023.12.038

16. Parretti H, Okosieme O, Vanderpump M. Current recommendations in the management of hypothyroidism: developed from a statement by the British Thyroid Association Executive. Br J Gen Pract. 2016;66(651):538-540. doi:10.3399/bjgp16X687493

17. Brenta G, Vaisman M, Sgarbi JA, Bergoglio LM, Andrada NC, Bravo PP, et al. Clinical practice guidelines for the management of hypothyroidism. Arq Bras Endocrinol Metabol. 2013;57(4):265-298. doi:10.1590/S0004-27302013000400003

18. Wiersinga WM, Duntas L, Fadeyev V, Nygaard B, Vanderpump MP. 2012 ETA guidelines: the use of L-T4 + L-T3 in the treatment of hypothyroidism. Eur Thyroid J. 2012;1(2):55-71. doi:10.1159/000339444

19. Jonklaas J, Bianco AC, Cappola AR, Celi FS, Fliers E, Heuer H, et al. Evidence-based use of levothyroxine/liothyronine combinations in treating hypothyroidism: a consensus document. Thyroid. 2021;31(2):156-182. doi:10.1089/thy.2020.0720

20. Endocrinology Branch, Chinese Medical Association. Guidelines for the diagnosis and treatment of adult hypothyroidism [in Chinese]. Chinese Journal of Endocrinology and Metabolism. 2017;33(2):167-180. doi:10.3760/cma.j.issn.1000-6699.2017.02.018

21. Chinese Medical Association, Chinese Medical Association Publishing House, General Practice Branch of Chinese Medical Association, et al. Guideline for primary care of hypothyroidism: practice version (2019) [in Chinese]. Chinese Journal of General Practitioners. 2019;18(11):1029-1033. doi:10.3760/cma.j.issn.1671-7368.2019.11.005

22. Chinese Medical Association, Clinical Pharmacy Branch of Chinese Medical Association, Chinese Medical Association Publishing House, et al. Guideline for rational medication of hypothyroidism in primary care [in Chinese]. Chinese Journal of General Practitioners. 2021;20(5):520-522. doi:10.3760/cma.j.cn114798-20210317-00250

23. Guyatt GH, Oxman AD, Vist GE, Kunz R, Falck-Ytter Y, Alonso-Coello P, et al. GRADE: an emerging consensus on rating quality of evidence and strength of recommendations. BMJ. 2008;336(7650):924-926. doi:10.1136/bmj.39489.470347.AD

## To do

- Confirm reference formatting in the Frontiers submission system.
