# Supplementary material for: Quality-recommendation paradox in adult hypothyroidism guidelines: a systematic appraisal of methodological quality, recommendation consistency, and citation impact
Source: Front Endocrinol (Lausanne). 2026 Oct 30;17:1998518. doi: 10.3389/fendo.2026.1998518 (PMC13759808; doi:10.3389/fendo.2026.1998518)
Supplement: Supplementary file 1 [file DataSheet1.zip › Supplementary_Reporting_S1_PRISMA_2020_Checklist.docx]

# **PRISMA 2020 Checklist**

| **Item #** | **Checklist item** | **Location where item is reported** |
| --- | --- | --- |
| 1 | Identify the report as a systematic review. | Title |
| 2 | See the PRISMA 2020 for Abstracts checklist (table 2). | Abstract |
| 3 | Describe the rationale for the review in the context of existing knowledge. | Section 1 (Introduction) |
| 4 | Provide an explicit statement of the objective(s) or question(s) the review addresses. | Section 1 (Introduction); Section 2.1 |
| 5 | Specify the inclusion and exclusion criteria for the review and how studies were grouped for the syntheses. | Section 2.3; Section 2.7 (subgroup grouping) |
| 6 | Specify all databases, registers, websites, organisations, reference lists and other sources searched or consulted to identify studies. Specify the date when each source was last searched or consulted. | Section 2.2; Supplementary File Search_Strategies.docx (PubMed, Web of Science, Scopus, CNKI, and VIP; last searched August 25, 2026; no registers or additional websites searched) |
| 7 | Present the full search strategies for all databases, registers and websites, including any filters and limits used. | Section 2.2; Supplementary File Search_Strategies.docx (full queries and record counts) |
| 8 | Specify the methods used to decide whether a study met the inclusion criteria of the review, including how many reviewers screened each record and each report retrieved, whether they worked independently, and if applicable, details of automation tools used in the process. | Section 2.4; Figure 1 (two independent reviewers; third-reviewer arbitration; no automation tools) |
| 9 | Specify the methods used to collect data from reports, including how many reviewers collected data from each report, whether they worked independently, any processes for obtaining or confirming data from study investigators, and if applicable, details of automation tools used in the process. | Section 2.5 (two independent reviewers; third-reviewer arbitration; no automation tools) |
| 10a | List and define all outcomes for which data were sought. Specify whether all results that were compatible with each outcome domain in each study were sought, and if not, the methods used to decide which results to collect. | Section 2.5; Sections 3.2-3.9 |
| 10b | List and define all other variables for which data were sought. Describe any assumptions made about any missing or unclear information. | Section 2.5 |
| 11 | Specify the methods used to assess risk of bias in the included studies, including details of the tool(s) used, how many reviewers assessed each study and whether they worked independently. | Not applicable. This systematic appraisal evaluated guidelines rather than primary studies; AGREE II domain scores served as the quality measure (Section 2.6). |
| 12 | Specify for each outcome the effect measure(s) used in the synthesis or presentation of results. | Not applicable. No intervention effects were synthesised. |
| 13a | Describe the processes used to decide which studies were eligible for each synthesis. | All 15 guidelines contributed to the quality and recommendation syntheses; citation anchoring included the 13 guidelines indexed by OpenAlex or Crossref (Sections 3.2-3.9). |
| 13b | Describe any methods required to prepare the data for presentation or synthesis, such as handling of missing summary statistics, or data conversions. | Section 2.5; Section 2.6 (standardized domain score calculation) |
| 13c | Describe any methods used to tabulate or visually display results of individual studies and syntheses. | Tables 1-2; Figures 2-6 |
| 13d | Describe any methods used to synthesise results and provide a rationale for the choice(s). If meta-analysis was performed, describe the model(s), method(s) to identify statistical heterogeneity, and software package(s) used. | Sections 2.6-2.8; descriptive synthesis and Spearman correlation; no meta-analysis; Python 3 |
| 13e | Describe any methods used to explore possible causes of heterogeneity among study results. | Not applicable. No quantitative synthesis was performed; heterogeneity was described narratively (Sections 3.4-3.7). |
| 13f | Describe any sensitivity analyses conducted to assess robustness of the synthesised results. | Not applicable. No meta-analysis was performed. |
| 14 | Describe any methods used to assess risk of bias due to missing results in a synthesis (arising from reporting biases). | Not applicable. No meta-analysis; missing citation data for two Chinese guidelines are addressed in Section 3.9 and Section 4 (Strengths and limitations). |
| 15 | Describe any methods used to assess certainty (or confidence) in the body of evidence for an outcome. | Not applicable. AGREE II appraises process and reporting quality; certainty of underlying evidence was not reassessed. |
| 16a | Describe the results of the search and selection process, from the number of records identified in the search to the number of studies included in the review, ideally using a flow diagram. | Section 3.1; Figure 1; Supplementary File PRISMA_Numbers.docx |
| 16b | Cite studies that might appear to meet the inclusion criteria, but which were excluded, and explain why they were excluded. | Section 3.1; Supplementary Table S1 (full-text exclusion ledger) |
| 17 | Cite each included study and present its characteristics. | Section 3.1; Table 1 |
| 18 | Present assessments of risk of bias for each included study. | Not applicable. No primary studies were included; AGREE II domain scores are presented in Table 1 and Figure 2. |
| 19 | For all outcomes, present, for each study: summary statistics and an effect estimate and its precision, ideally using structured tables or plots. | Not applicable. The appraisal unit was the guideline; results are presented at guideline level (Tables 1-2; Figures 2-6). |
| 20a | For each synthesis, briefly summarise the characteristics and risk of bias among contributing studies. | Not applicable. No meta-analysis; narrative synthesis is summarized in Sections 3.4-3.9. |
| 20b | Present results of all statistical syntheses conducted. If meta-analysis was done, present for each the summary estimate and its precision and measures of statistical heterogeneity. | Not applicable. No meta-analysis was performed. |
| 20c | Present results of all investigations of possible causes of heterogeneity among study results. | Not applicable. No quantitative synthesis was performed. |
| 20d | Present results of all sensitivity analyses conducted to assess the robustness of the synthesised results. | Not applicable. No meta-analysis was performed. |
| 21 | Present assessments of risk of bias due to missing results (arising from reporting biases) for each synthesis assessed. | Not applicable. No meta-analysis was performed. |
| 22 | Present assessments of certainty (or confidence) in the body of evidence for each outcome assessed. | Not applicable. AGREE II does not assess certainty of recommendations; this is acknowledged in Section 4 (Strengths and limitations). |
| 23a | Provide a general interpretation of the results in the context of other evidence. | Section 4 (Discussion) |
| 23b | Discuss any limitations of the evidence included in the review. | Section 4 (Strengths and limitations) |
| 23c | Discuss any limitations of the review processes used. | Section 4 (Strengths and limitations) |
| 23d | Discuss implications of the results for practice, policy, and future research. | Section 4; Section 5 (Conclusion) |
| 24a | Provide registration information for the review, including register name and registration number, or state that the review was not registered. | Section 2.1; PROSPERO CRD420261487294 |
| 24b | Indicate where the review protocol can be accessed, or state that a protocol was not prepared. | Section 2.1; PROSPERO record (CRD420261487294) |
| 24c | Describe and explain any amendments to information provided at registration or in the protocol. | No formal PROSPERO amendment was submitted. Post-registration deviations were as follows: the executed search used PubMed, Web of Science, Scopus, CNKI, and VIP, whereas the registered plan named PubMed, supplementary guideline websites, CNKI, and Wanfang; database access determined the final source set, and Embase remained unavailable. The LT4/LT3 Consensus 2021 was included because it was a systematically developed standard document addressing an explicitly contested question. Version-evolution and citation-impact analyses were added as clearly labelled exploratory secondary analyses. See Sections 2.2, 2.3, 3.5, 3.9, and Strengths and limitations. |
| 25 | Describe sources of financial or non-financial support for the review, and the role of the funders or sponsors in the review. | Section Funding |
| 26 | Declare any competing interests of review authors. | Section Conflict of interest |
| 27 | Report which of the following are publicly available and where they can be found: template data collection forms; data extracted from included studies; data used for all analyses; analytic code; any other materials used in the review. | Section Data availability statement; raw AGREE II rating sheets are available from the corresponding author on reasonable request |
