# Supplementary material for: Quality-recommendation paradox in adult hypothyroidism guidelines: a systematic appraisal of methodological quality, recommendation consistency, and citation impact
Source: Front Endocrinol (Lausanne). 2026 Oct 30;17:1998518. doi: 10.3389/fendo.2026.1998518 (PMC13759808; doi:10.3389/fendo.2026.1998518)
Supplement: Supplementary file 1 [file DataSheet1.zip › Supplementary_Reporting_S2_PRISMA-S_Checklist.docx]

# **PRISMA-S Checklist**

| **Item #** | **Checklist item** | **Location where item is reported** |
| --- | --- | --- |
| 1 | Name each individual database searched, stating the platform for each. | Section 2.2; Supplementary File Search_Strategies.docx (PubMed via NCBI E-utilities; Web of Science All Databases; Scopus; CNKI; VIP) |
| 2 | If databases were searched simultaneously on a single platform, state the name of the platform, listing all of the databases searched. | Not applicable. Each database was searched separately on its own platform. |
| 3 | List any study registries searched. | Not applicable. No study registries were searched (guideline appraisal). |
| 4 | Describe any online or print source purposefully searched or browsed, and how this was done. | Not applicable. No additional online or print sources were browsed. |
| 5 | Indicate whether cited references or citing references were examined, and describe any methods used for locating cited/citing references. | Not performed for study identification. Citation counts for the exploratory impact analysis were retrieved through OpenAlex and Crossref. |
| 6 | Indicate whether additional studies or data were sought by contacting authors, experts, manufacturers, or others. | Not performed. |
| 7 | Describe any additional information sources or search methods used. | None. |
| 8 | Include the search strategies for each database and information source, copied and pasted exactly as run. | Supplementary File Search_Strategies.docx |
| 9 | Specify that no limits were used, or describe any limits or restrictions applied to a search and provide justification for their use. | Section 2.2; Supplementary File Search_Strategies.docx. The date range was limited to January 1, 2010 to August 25, 2026 to capture contemporary guidance; language was unrestricted during searching, with only Chinese and English documents proceeding to screening. No other database limits or filters were applied. |
| 10 | Indicate whether published search filters were used, and if so, cite the filter(s) used. | No published search filters were used. |
| 11 | Indicate when search strategies from other literature reviews were adapted or reused for a substantive part or all of the search, citing the previous review(s). | No search strategies from other reviews were adapted or reused. |
| 12 | Report the methods used to update the search(es). | No updates; each database was searched once on August 25, 2026. |
| 13 | For each search strategy, provide the date when the last search occurred. | August 25, 2026 (all databases); see Supplementary File Search_Strategies.docx |
| 14 | Describe any search peer review process. | The search strategies were checked by a second team member, and the executed searches were verified on August 26, 2026; a formal PRESS review was not performed. See Supplementary File Search_Strategies.docx. |
| 15 | Document the total number of records identified from each database and other information sources. | Section 2.2; Figure 1 (295 records: PubMed 113, Web of Science 72, Scopus 69, CNKI 21, VIP 20) |
| 16 | Describe the processes and any software used to deduplicate records from multiple database searches and other information sources. | Section 2.4; Figure 1; Supplementary Files Search_Strategies.docx and PRISMA_Numbers.docx (71 duplicate English records removed; 224 records remained after deduplication: 183 English and 41 Chinese; DOI/PMID-based for English records, title/author/year for Chinese records) |
