# Supplementary material for: Quality-recommendation paradox in adult hypothyroidism guidelines: a systematic appraisal of methodological quality, recommendation consistency, and citation impact
Source: Front Endocrinol (Lausanne). 2026 Oct 30;17:1998518. doi: 10.3389/fendo.2026.1998518 (PMC13759808; doi:10.3389/fendo.2026.1998518)
Supplement: Supplementary file 1 [file DataSheet1.zip › Supplementary_Reporting_S4_Search_Strategies.docx]

# Search Strategies and Execution Record

**Formal search date:** August 25, 2026

**Databases:** PubMed, Web of Science, Scopus, CNKI, and VIP

**Date range:** January 1, 2010 to August 25, 2026

**Language:** not restricted during the search; only Chinese and English documents proceeded to screening

**Embase:** not performed because institutional access was unavailable

## PubMed (platform: NCBI E-utilities)

Search date: 2026-08-25. Records identified: 113.

("Hypothyroidism"[MeSH] OR "hypothyroidism"[tiab] OR "hypothyroid"[tiab] OR "subclinical hypothyroidism"[tiab] OR "myxedema"[tiab] OR "myxoedema"[tiab]) AND (guideline[pt] OR "practice guideline"[pt] OR "clinical practice guideline"[tiab] OR "clinical practice guidelines"[tiab] OR "practice guidelines"[tiab]) AND ("2010/01/01"[dp]:"2026/08/25"[dp])

## Web of Science (platform: Web of Science All Databases)

Search date: 2026-08-25. Records identified: 72.

TS=("hypothyroidism" OR "hypothyroid" OR "subclinical hypothyroidism" OR "myxedema" OR "myxoedema") AND TS=(guideline OR "practice guideline" OR "clinical practice guideline" OR "practice guidelines") AND PY=2010-2026

## Scopus

Search date: 2026-08-25. Records identified: 69.

TITLE-ABS-KEY("hypothyroidism" OR "hypothyroid" OR "subclinical hypothyroidism" OR "myxedema" OR "myxoedema") AND TITLE-ABS-KEY(guideline OR "practice guideline" OR "clinical practice guideline" OR "practice guidelines") AND PUBYEAR > 2009 AND PUBYEAR < 2027

## CNKI

Search date: 2026-08-25. Records identified: 21.

The CNKI interface was used with title-field searches (Chinese interface; queries shown exactly as entered).

```
篇名：甲状腺功能减退症 AND 篇名：指南   (title: hypothyroidism AND title: guideline)   16 records
篇名：甲状腺功能减退症 AND 篇名：共识   (title: hypothyroidism AND title: consensus)   4 records
篇名：左甲状腺素 AND 篇名：指南        (title: levothyroxine AND title: guideline)    1 record
```

## VIP

Search date: 2026-08-25. Records identified: 20.

```
篇名：甲减 AND 篇名：指南   (title: hypothyroidism AND title: guideline)   16 records
篇名：甲减 AND 篇名：共识   (title: hypothyroidism AND title: consensus)   4 records
```

## Deduplication

English records were deduplicated by DOI and PMID, retaining the PubMed version. Chinese records were deduplicated by title, author, and year. After deduplication, 224 records remained (183 English and 41 Chinese), and 80 records proceeded to full-text assessment.

## Executors

Searches were performed and verified by the study team (verification registered on August 26, 2026).
