# Supplementary material for: Quality-recommendation paradox in adult hypothyroidism guidelines: a systematic appraisal of methodological quality, recommendation consistency, and citation impact
Source: Front Endocrinol (Lausanne). 2026 Oct 30;17:1998518. doi: 10.3389/fendo.2026.1998518 (PMC13759808; doi:10.3389/fendo.2026.1998518)
Supplement: Supplementary file 1 [file DataSheet1.zip › Supplementary_Reporting_S3_PRISMA_Numbers.docx]

# PRISMA 2020 flow diagram numbers (final, 2026-08-26)

This file documents the numbers used to draw the PRISMA flow diagram. The record was reviewed and corresponds to 40_PRISMA_ScreeningRecords.csv.

## 1. Identification

| **Source** | **Records** |
| --- | --- |
| PubMed (formal search 2026-08-25) | 113 |
| Web of Science | 72 |
| Scopus | 69 |
| CNKI | 21 |
| VIP | 20 |
| Embase | 0 (not performed) |
| Total | 295 |

## 2. Screening and eligibility

- English databases yielded 254 records; after two-step title deduplication (files 42 and 44), 183 remained, with 71 duplicates removed.
- Chinese databases yielded 41 records, handled separately with no overlap with English records.
- Records after deduplication: 224 (English 183 + Chinese 41).
- Records excluded at title/abstract screening: 144 (English 108 + Chinese 36).
- Records remaining after screening: 80 (English 75 + Chinese 5).
- Records assessed in full text: 80.
- Records excluded in full text: 65, comprising 27 full-text review exclusions (file 46), 30 candidate refinement exclusions (files 46 to 47), and 8 final exclusions (file 50).
- Guidelines included: 15 (English 12 + Chinese 3), file 52.

## 3. Traceability

| **Stage** | **Count** | **Source file** |
| --- | --- | --- |
| Database hits | 113 / 72 / 69 / 21 / 20 | Files 25, 29, 43, 28 |
| English deduplication | 183 | Files 42, 44 |
| Title/abstract exclusions | 108 (English) | File 45 |
| Candidates | 75 (English) + 5 (Chinese) | Files 45, 46, 28 |
| Full-text assessment | 80 | Files 45, 28 |
| Full-text exclusions | 65 | Files 46, 47, 50 |
| Included | 15 | File 52 |

## 4. Notes

1. Embase was not performed and is excluded from the total.

2. The 36 Chinese title/abstract exclusions are the difference between 41 and 5; categories are in file 28.

3. The 30 candidate refinement exclusions are a stage-level difference (48 kept in file 46 minus 18 candidates in file 47); the full 65-row ledger is available as Supplementary Table S1.
