# Supplementary material for: Quality-recommendation paradox in adult hypothyroidism guidelines: a systematic appraisal of methodological quality, recommendation consistency, and citation impact
Source: Front Endocrinol (Lausanne). 2026 Oct 30;17:1998518. doi: 10.3389/fendo.2026.1998518 (PMC13759808; doi:10.3389/fendo.2026.1998518)
Supplement: Supplementary file 1 [file DataSheet1.zip › Supplementary_File_S3_AGREE_II_Rating_Form.docx]

# AGREE II Rating Form Used in This Study

This form documents the 23 AGREE II items and the 1-7 rating scale used by the four independent raters. The instrument was applied as published and is cited as reference 6 in the manuscript. The AGREE II instrument is available from the AGREE Research Trust.

Citation: Brouwers MC, Kho ME, Browman GP, Burgers JS, Cluzeau F, Feder G, et al. AGREE II: advancing guideline development, reporting and evaluation in health care. CMAJ. 2010;182(18):E839-E842. doi:10.1503/cmaj.090449.

Rating scale: 1 = strongly disagree; 2 = disagree; 3 = disagree somewhat; 4 = neither agree nor disagree; 5 = agree somewhat; 6 = agree; 7 = strongly agree.

| **Domain** | **Item** | **AGREE II item** | **Rating scale** |
| --- | --- | --- | --- |
| D1 | 1 | The overall objective(s) of the guideline is/are specifically described. | 1-7 |
| D1 | 2 | The health question(s) covered by the guideline is/are specifically described. | 1-7 |
| D1 | 3 | The population to whom the guideline is meant to apply is specifically described. | 1-7 |
| D2 | 4 | The guideline development group includes individuals from all relevant professional groups. | 1-7 |
| D2 | 5 | The views and preferences of the target population have been sought. | 1-7 |
| D2 | 6 | The target users of the guideline are clearly defined. | 1-7 |
| D3 | 7 | Systematic methods were used to search for evidence. | 1-7 |
| D3 | 8 | The criteria for selecting the evidence are clearly described. | 1-7 |
| D3 | 9 | The strengths and limitations of the body of evidence are clearly described. | 1-7 |
| D3 | 10 | The methods for formulating the recommendations are clearly described. | 1-7 |
| D3 | 11 | The health benefits, side effects, and risks have been considered in formulating the recommendations. | 1-7 |
| D3 | 12 | There is an explicit link between the recommendations and the supporting evidence. | 1-7 |
| D3 | 13 | The guideline has been externally reviewed by experts prior to its publication. | 1-7 |
| D3 | 14 | A procedure for updating the guideline is provided. | 1-7 |
| D4 | 15 | The recommendations are specific and unambiguous. | 1-7 |
| D4 | 16 | The different options for management of the condition or health issue are clearly presented. | 1-7 |
| D4 | 17 | Key recommendations are easily identifiable. | 1-7 |
| D5 | 18 | The guideline describes facilitators and barriers to its application. | 1-7 |
| D5 | 19 | The guideline provides advice and/or tools on how the recommendations can be put into practice. | 1-7 |
| D5 | 20 | The potential resource implications of applying the recommendations have been considered. | 1-7 |
| D5 | 21 | The guideline presents monitoring and/or auditing criteria. | 1-7 |
| D6 | 22 | The views of the funding body have not influenced the content of the guideline. | 1-7 |
| D6 | 23 | Competing interests of guideline development group members have been recorded and addressed. | 1-7 |

Domain score formula: (obtained score - minimum possible score) / (maximum possible score - minimum possible score) x 100%.

Overall assessment: each rater also assigned an overall quality score from 1 to 7. The guideline-level overall score was the mean of the four raters' overall scores.

Variation from the published instrument: none. The formula and the 23-item structure were applied without modification.
