# Supplementary material for: Quality-recommendation paradox in adult hypothyroidism guidelines: a systematic appraisal of methodological quality, recommendation consistency, and citation impact
Source: Front Endocrinol (Lausanne). 2026 Oct 30;17:1998518. doi: 10.3389/fendo.2026.1998518 (PMC13759808; doi:10.3389/fendo.2026.1998518)
Supplement: Supplementary file 1 [file DataSheet1.zip › Supplementary_File_S4_Statistical_Analysis_and_Verification.docx]

# Statistical Analysis and Verification Report

This report documents the calculations underlying the standardized AGREE II scores, overall quality scores, inter-rater reliability estimates, and exploratory citation correlation reported in the manuscript. All calculations were repeated from the four de-identified rater workbooks.

## Data Sources and Handling

Raw data: four independent rater workbooks containing 23 item scores for each of the 15 included guidelines.

Missing values: no item-level scores were missing among the included guidelines.

Software: Python 3 using standard mathematical and CSV libraries; all computations were independently reproducible from the supplied raw item scores.

## AGREE II Score Calculation

Domain score = (obtained score - minimum possible score) / (maximum possible score - minimum possible score) x 100%.

Minimum possible score = number of items in the domain x number of raters x 1. Maximum possible score = number of items in the domain x number of raters x 7.

Guideline-level overall quality score = mean of each rater's mean score across all 23 items.

## Standardized Domain and Overall Quality Scores

| **Guideline** | **Overall** | **D1** | **D2** | **D3** | **D4** | **D5** | **D6** |
| --- | --- | --- | --- | --- | --- | --- | --- |
| BMJ 2019 | 6.25 | 87.5 | 84.7 | 90.6 | 86.1 | 88.5 | 79.2 |
| AACE/ATA 2012 | 6.2 | 86.1 | 79.2 | 90.6 | 86.1 | 89.6 | 77.1 |
| ETA 2025 | 6.1 | 90.3 | 76.4 | 86.5 | 86.1 | 86.5 | 79.2 |
| ATA 2014 | 5.92 | 87.5 | 73.6 | 83.3 | 83.3 | 82.3 | 77.1 |
| ETA 2018 (central hypothyroidism) | 5.85 | 87.5 | 77.8 | 81.2 | 83.3 | 82.3 | 66.7 |
| Italian 2025 | 5.83 | 84.7 | 75.0 | 80.2 | 81.9 | 82.3 | 79.2 |
| ETA 2013 | 5.76 | 86.1 | 72.2 | 78.1 | 81.9 | 84.4 | 70.8 |
| ASRM 2024 | 5.58 | 79.2 | 79.2 | 75.0 | 77.8 | 79.2 | 66.7 |
| Brazilian 2013 | 5.37 | 77.8 | 66.7 | 73.4 | 76.4 | 70.8 | 70.8 |
| CMA 2017 | 5.29 | 87.5 | 65.3 | 69.8 | 76.4 | 66.7 | 66.7 |
| LT4/LT3 Consensus 2021 | 5.22 | 81.9 | 56.9 | 70.3 | 79.2 | 66.7 | 66.7 |
| ETA 2012 (L-T4+L-T3) | 5.15 | 81.9 | 52.8 | 69.8 | 75.0 | 72.9 | 56.2 |
| BTA 2016 | 5.09 | 81.9 | 52.8 | 62.0 | 79.2 | 74.0 | 66.7 |
| Chinese Primary Care 2019 | 4.86 | 76.4 | 47.2 | 62.0 | 73.6 | 65.6 | 64.6 |
| Chinese Primary Care Medication 2021 | 4.77 | 68.1 | 56.9 | 61.5 | 65.3 | 63.5 | 64.6 |

## Inter-Rater Reliability

Model: two-way random-effects, absolute-agreement intraclass correlation coefficient. ICC(2,1) is the single-measure coefficient and ICC(2,k) is the average-measure coefficient across four raters.

| **Domain** | **Items** | **ICC(2,1)** | **ICC(2,k)** |
| --- | --- | --- | --- |
| D1 | 45 | 0.318 | 0.651 |
| D2 | 45 | 0.68 | 0.895 |
| D3 | 120 | 0.567 | 0.84 |
| D4 | 45 | 0.348 | 0.681 |
| D5 | 60 | 0.547 | 0.828 |
| D6 | 30 | 0.417 | 0.741 |

## Exploratory Citation Correlation

Citation counts were available for 13 of 15 guidelines. The tie-corrected Spearman rank correlation between overall quality and citation count was rho = 0.368.

Citation counts were strongly influenced by publication age, journal visibility, and clinical controversy. This analysis was exploratory, descriptive, and not interpreted causally.

## Verification Statement

All reported domain scores, overall quality scores, ICC values, and the Spearman correlation were reproduced from the raw item-level ratings. The consolidated item-level dataset is provided as AGREE_II_Item_Scores.csv. No results were imputed or replaced. Independent statistical review is welcome.
