# Supplementary material for: Quality-recommendation paradox in adult hypothyroidism guidelines: a systematic appraisal of methodological quality, recommendation consistency, and citation impact
Source: Front Endocrinol (Lausanne). 2026 Oct 30;17:1998518. doi: 10.3389/fendo.2026.1998518 (PMC13759808; doi:10.3389/fendo.2026.1998518)
Supplement: Supplementary file 1 [file DataSheet1.zip › Supplementary_Index_S1_File_Index.docx]

# Supplementary File Index

Final supplementary package. Each data set is provided in one file format only to avoid duplicate submissions during production.

| **Code** | **File** | **Purpose** |
| --- | --- | --- |
| S1 | Supplementary_Table_S1_FullTextExclusion_Ledger.csv | Full-text exclusion ledger for the 65 excluded records. |
| S2 | Supplementary_Table_S2_Disconnect_Matrix.csv | Content-methodology disconnect matrix. |
| S3 | Supplementary_File_S3_AGREE_II_Rating_Form.docx | AGREE II rating form with 23 items, six domains, and 1-7 scale. |
| S4 | Supplementary_File_S4_Statistical_Analysis_and_Verification.docx | Statistical analysis, calculation rules, and verification results. |
| Data S1 | Supplementary_Data_S1_AGREE_II_Item_Scores.csv | Item-level AGREE II ratings from four raters. |
| Data S2 | Supplementary_Data_S2_IncludedGuidelines.csv | Characteristics of the 15 included guidelines. |
| Data S3 | Supplementary_Data_S3_ReScore_Summary.csv | Standardized domain scores and overall quality scores. |
| Data S4 | Supplementary_Data_S4_ReScore_ICC.csv | Domain-specific ICC estimates. |
| Data S5 | Supplementary_Data_S5_Recommendation_Extraction.csv | Structured extraction of six contested recommendation topics. |
| Data S6 | Supplementary_Data_S6_Recommendation_ConflictMatrix.csv | Recommendation consistency matrix. |
| Data S7 | Supplementary_Data_S7_VersionEvolution.csv | Version-evolution mapping for ETA and Chinese guideline series. |
| Data S8 | Supplementary_Data_S8_EndoCompass_Coverage.csv | Emerging-topic coverage classifications. |
| Reporting S1 | Supplementary_Reporting_S1_PRISMA_2020_Checklist.docx | PRISMA 2020 checklist. |
| Reporting S2 | Supplementary_Reporting_S2_PRISMA-S_Checklist.docx | PRISMA-S checklist. |
| Reporting S3 | Supplementary_Reporting_S3_PRISMA_Numbers.docx | PRISMA flow-diagram numbers and traceability. |
| Reporting S4 | Supplementary_Reporting_S4_Search_Strategies.docx | Search strategies and execution record. |
| Reporting S5 | Supplementary_Reporting_S5_PRISMA_ScreeningRecords.csv | Screening numbers used in the PRISMA flow diagram. |
| Reporting S6 | Supplementary_Reporting_S6_References.docx | Reference file. |
| Index S1 | Supplementary_Index_S1_File_Index.docx | Index of all supplementary files. |
